# Testicular expression of long non–coding RNAs is affected by curative GnRHa treatment of cryptorchidism

**DOI:** 10.1186/s12610-019-0097-3

**Published:** 2019-12-27

**Authors:** Faruk Hadziselimovic, Gilvydas Verkauskas, Beata Vincel, Michael B. Stadler

**Affiliations:** 1 Cryptorchidism Research Institute, Children’s Day Care Center, 4410 Liestal, Switzerland; 2 Children’s Surgery Centre, Faculty of Medicine, Vilnius University, 01513 Vilnius, Lithuania; 3 Children’s Surgery Centre, Clinic of Gastroenterology, Nephrourology and Surgery, Institute of Clinical Medicine, Faculty of Medicine, Vilnius University, Vilnius, Lithuania; 4 Friedrich Miescher Institute for Biomedical Research, Basel, Switzerland; 5 Swiss Institute of Bioinformatics, Basel, Switzerland

**Keywords:** Spermatogenesis, Cryptorchidism, Male infertility, GnRHa, *lncRNAs*, Antisense *lncRNAs*, *lincRNAs*, Mitosis, Spermatogénèse, cryptorchidie, GnRHa, *ARNlnc*, *ARNlnc* antisense, mitose

## Abstract

**Background:**

Cryptorchidism is a frequent endocrinopathy in boys that has been associated with an increased risk of developing testicular cancer and infertility. The condition is curable by combined surgery and hormonal treatment during early pre-pubertal stages using gonadotropin releasing hormone agonist (GnRHa). However, whether the treatment also alters the expression of testicular long non-coding RNAs (lncRNAs) is unknown. To gain insight into the effect of GnRHa on testicular lncRNA levels, we re-analyzed an expression dataset generated from testicular biopsies obtained during orchidopexy for bilateral cryptorchidism.

**Results:**

We identified *EGFR-AS1*, *Linc-ROR*, *LINC00221, LINC00261, LINC00282, LINC00293, LINC00303, LINC00898, LINC00994, LINC01121, LINC01553,* and *MTOR-AS1* as potentially relevant for the stimulation of cell proliferation mediated by GnRHa based on their direct or indirect association with rapidly dividing cells in normal and pathological tissues. Surgery alone failed to alter the expression of these transcripts.

**Conclusion:**

Given that lncRNAs can cooperate with chromatin-modifying enzymes to promote epigenetic regulation of genes, GnRHa treatment may act as a surrogate for mini-puberty by triggering the differentiation of Ad spermatogonia via lncRNA-mediated epigenetic effects. Our work provides additional molecular evidence that infertility and azoospermia in cryptorchidism, resulting from defective mini-puberty cannot be cured with successful orchidopexy alone.

## Résumé

**Contexte:**

La cryptorchidie est. une endocrinopathie fréquente chez les garçons. Elle est. associée à un risque élevé de cancer des testicules et d’infertilité. La cryptorchidie peut être soignée par une thérapie incluant une intervention chirurgicale et un traitement hormonal par l’agoniste de l’hormone GnRH. Alors que l’effet de la thérapie sur l’expression des ARNm a été analysé, ses conséquences pour la transcription des longs ARNs non codants (ARNlnc) testiculaires restent inconnues. Afin de mieux comprendre les effets du GnRHa sur les concentrations cellulaires des ARNlnc dans le testicule, nous avons analysé des données d’expression d’ARN par séquençage (ARN-Seq) générées en utilisant des biopsies testiculaires obtenues dans le cadre d’une orchidopexie pour cryptorchidie bilatérale.

**Résultats:**

Nous avons identifié les *ARNlnc EGFR-AS1, Linc-ROR*, *LINC00221, LINC00261, LINC00282, LINC00293, LINC00303, LINC00898, LINC00994, LINC01121, LINC01553*, et *MTOR-AS1* comme potentiellement pertinents pour la stimulation de la prolifération cellulaire induite par le GnRHa. Cette conclusion fait référence à leur association directe ou indirecte avec la croissance et division cellulaire mitotique rapide dans les tissus normaux et pathologiques. Nous constatons également que la chirurgie seule n’a pas d’effet détectable par ARN-Seq sur l’expression de ces ARNlnc.

**Conclusion:**

Étant donné que certains ARNlnc coopèrent avec des enzymes ayant un effet sur la structure chromatinienne et la régulation épigénétique des gènes, le traitement par GnRHa pourrait substituer la mini-puberté en déclenchant la différenciation des spermatogonies Ad par un mécanisme épigénétiques qui dépendrait des ARNlnc. Notre travail révèle des nouvelles pistes moléculaires soutenant l’hypothèse que l’infertilité et l’azoospermie associées avec la cryptorchidie sont la conséquence d’une anomalie de la mini-puberté. Cela explique pourquoi une thérapie efficace de cette pathologie ne nécessite pas seulement l’orchidopexie mais aussi un traitement hormonal.

## Introduction

Long non-coding RNAs (lncRNAs) have emerged as key regulators of gene expression in embryonic stem-cell (ESC) self-renewal and differentiation. In ESCs, lncRNAs are regulated at the genetic level by transcription factor binding to lncRNA gene promoters. A major function of lncRNAs is the regulation of specific gene expression at multiple steps, including the recruitment and expression of basal transcription machinery, post-transcriptional modifications, and epigenetics [1]. LncRNAs have also been proposed to play a targeting role by binding to certain methyltransferases and demethylases, and directing them to specific genomic locations. Depending on the biological context, certain methylation events are stably maintained (e.g., methylation involved in inheritance through mitosis of a silenced heterochromatin state), whereas others have to be amenable to change (e.g., when cells differentiate or respond to environmental cues) [2–5]. The so-called natural antisense transcripts (NATs) have been shown to regulate gene expression by affecting transcription and mRNA stability [2–5]. Almost 80% of the mammalian genome is transcribed, and many genomic loci produce RNAs from both sense and antisense DNA strands [6–8], though the functional importance of most of these transcripts is only poorly characterized.

We have previously demonstrated that the presence of type A dark (Ad) spermatogonia in the testis is a marker of low infertility risk (LIR), whereas low or absent levels (below a critical threshold) indicate high infertility risk (HIR) [9, 10]. Treatment with a gonadotropin-releasing hormone agonist (GnRHa, buserelin) enables the Ad spermatogonia population to recover, significantly improving fertility in HIR patients [11]. GnRHa induces a broad transcriptional response, including genes encoding proteins involved in pituitary development, the hypothalamic-pituitary-gonadal axis, and testosterone synthesis [12]. Earlier work focused on protein-coding mRNAs; consequently, nothing is known about the expression of lncRNAs in the treatment of cryptorchidism.

We identified several hundred GnRHa-responsive lncRNAs, which were grouped into long intergenic non-coding RNAs (lincRNAs) and antisense (AS) lncRNAs. We selected candidates on the basis of their expression profiles and then prioritized them for roles in cell growth, differentiation, and disease based on a literature search in PubMed (www.ncbi.nlm.nih.gov/pubmed/). We also included in this search protein-coding genes located upstream or downstream lincRNAs and sense genes overlapping AS-lncRNAs. In addition, we explored the RNA-RNA interaction data available in the RISE database (http://rise.life.tsinghua.edu.cn). Finally, we interpreted lncRNA expression signals in the context of protein/RNA profiling data published by the Human Protein Atlas (www.proteinatlas.org) and RNA-sequencing data from HIR/LIR patients [12]. We propose that certain hormone-responsive lncRNAs may play a role in establishing adult spermatogenesis during pre-pubertal stages of development by controlling testicular cell proliferation.

## Materials and methods

### Study population and biopsy sample collection

**Fig. 1:**
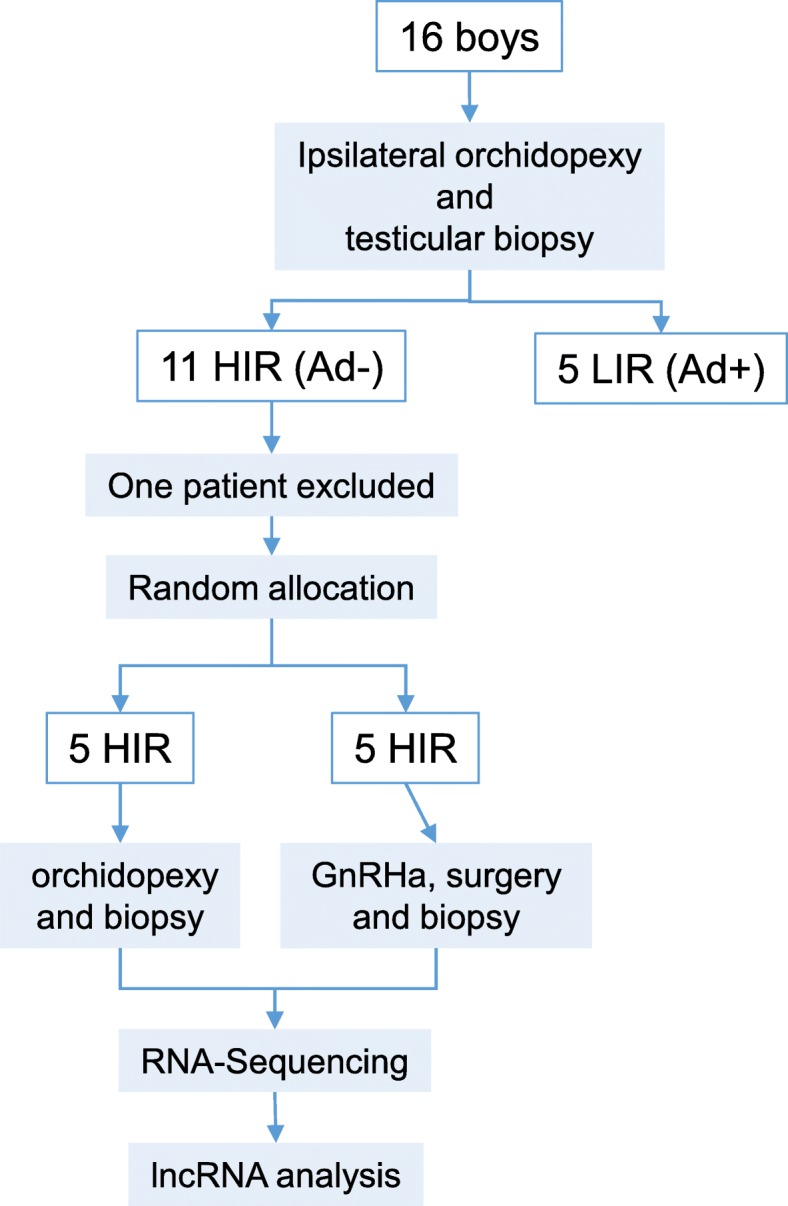
Flow chart of the study design and the selection of patients and samples for RNA-sequencing based on expression profiling**.** High infertility risk (HIR) and low infertility risk (LIR) patients are indicated. Classification is based on the absence (Ad-) or presence (Ad+) of Ad spermatogonia

The samples used in this study have been described elsewhere [12–14]. A cryptorchid testis is defined as a testis localized outside of the scrotum and incapable of being brought into a stable scrotal position. Sixteen boys with isolated bilateral cryptorchidism who underwent orchidopexy were prospectively included in this study (Fig. 1). The patients had a median age of 18.5 months (range 8–59 months). During the first orchidopexy, biopsies of the ipsilateral testicle were obtained from all patients. Based on histological evaluation, biopsies were categorized into two groups, Ad- (or HIR) and Ad+ (or LIR),. The Ad- group included biopsies with no Ad, and the Ad+ group included testes with Ad spermatogonia (Fig. 1). Cryptorchid boys in the Ad- group had 8-times lower plasma LH levels (0.11 IU/L) than the Ad+ group (0.89 IU/L, *p* < 0.009), indicating hypogonadotropic hypogonadism [13]. Five boys (Ad- group) were randomly included in each arm. One HIR patients was excluded (Fig. 1.) In the GnRHa-treated group, the median total germ cell count per tubule (S/T) increased from 0.11 to 0.42 (*p* = 0.03, paired-samples Wilcoxon test, one-tailed). In the surgery only group, the median S/T did not change and none of the testes had Ad spermatogonia. In contrast, in the GnRHa treated group, all testes completed the transition from gonocytes to Ad spermatogonia (*p* = 0.008; Fisher test, 2-tailed) [14].

### RNA-sequencing data analysis

The workflow from RNA isolation to purification, library preparation, sequencing, data analysis, and expression level analysis has been described in detail elsewhere [12]. Determination of differentially expressed genes, statistical analyses, and model design were carried out as described previously [12]. Genomic coordinates for known lncRNAs were obtained from the Bioconductor package TxDb.Hsapiens.UCSC.hg19.lincRNAsTranscripts (version 3.2.2). Only genes with at least one read per million, in at least two samples were included. *P*-values and fold-changes were calculated for the treatment factor, and differentially expressed genes were defined as those with a false discovery rate (FDR) of less than 0.05. Raw data files are available at the Database of Genotypes and Phenotypes (dbGaP) under accession number phs001275.v1.p1. Expression signals are given in standard RKPM units. They are calculated as follows: the number of reads mapped to a gene sequence is divided by the length of the gene sequence over 1000 multiplied by the total number of mapped reads per sample over 1′000’000.

### LncRNA data interpretation

We analyzed lincRNA and AS-lncRNA expression in cryptorchid patients with HIR before and after GnRH treatment to identify all RNAs annotated as antisense (AS) transcripts, as well as all RNAs annotated as lincRNAs with logFC > 1.0. In addition, we compared lincRNA and AS- lncRNA expression between the HIR and LIR groups of cryptorchid patients and analyzed those with lower expression in the HIR group. LincRNAs and AS- lncRNAs were prioritized based on RNA-RNA interactions, revealing the lncRNAs, AS- lncRNAs, or mRNAs encoding proteins involved in spermatogenesis or fertility, and important lncRNAs or mRNAs encoding proteins involved in cell division/growth, signaling pathways, and cancer. Furthermore, we included five lincRNA directly related to spermatogenesis that had a log FC > 1.0. After the AS- lncRNA candidates were identified, they were prioritized based on a PubMed literature search of themselves and their overlapping sense mRNA/protein, revealing roles in spermatogenesis, fertility, cell division/growth, signaling pathways, and cancer (www.ncbi.nlm.nih.gov/pubmed). The RNA annotation was verified using Ensembl (www.ensembl.org; release 97). The lincRNA/mRNA expression was interpreted using GermOnline (www.germonline.org; version 4.0), Human Protein Atlas (www.proteinatlas.org; version 18), and Genevestigator (www.genevestigator.com; version 7.3.1). Experimentally validated RNA-RNA interaction data were retrieved from RISE (http://rise.life.tsinghua.edu.cn; version 1.0).

## Results

### Global effects on testicular lncRNA levels in response to GnRHa treatment

**Fig. 2:**
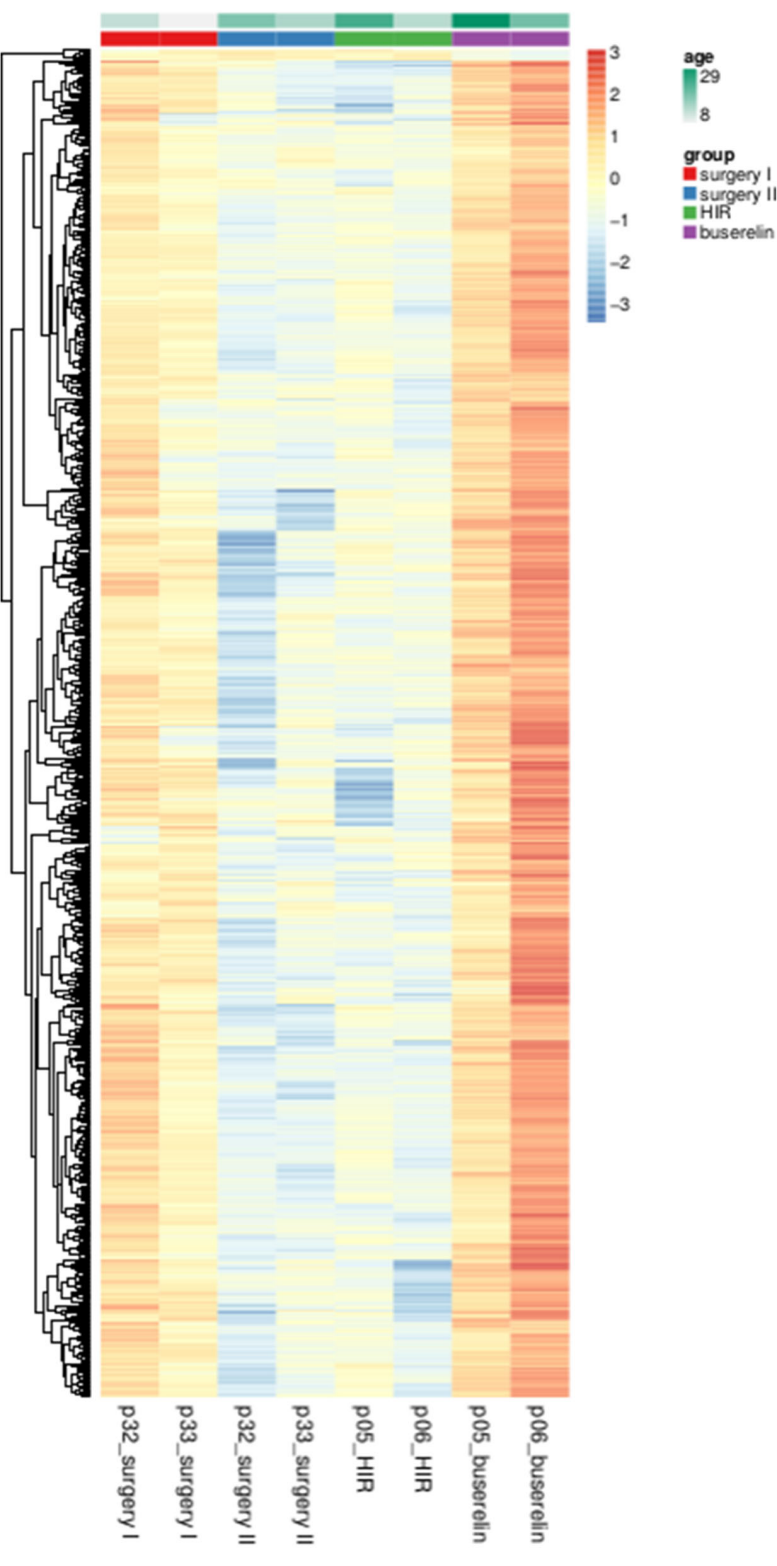
RNA-sequencing data for lncRNAs. A false color heatmap (red is high, blue is low) shows data from pairs of testes, analyzed. Horizontal bars at the top indicate patient categories and age. Four sample from two HIR patients (p32 and p33; biopsy number) having had surgery “only” treatment; line 1 and 2 during first surgery obtained from ipsilateral testis (I), line 3 and 4 show results from contralateral testis six months after first orchidopexy (II) Buserelin treated patients p5 and p6; line 5 an 6 before treatment and line 7 and 8 contralateral testis after hormonal treatment. Color scales for expression (red/blue) and age (green) are shown

First, we identified significantly differentially expressed lncRNAs in duplicate testicular biopsies from LIR and HIR patients who underwent surgical correction of undescended testis (Fig. 2, lanes 1–4).

**Fig. 3:**
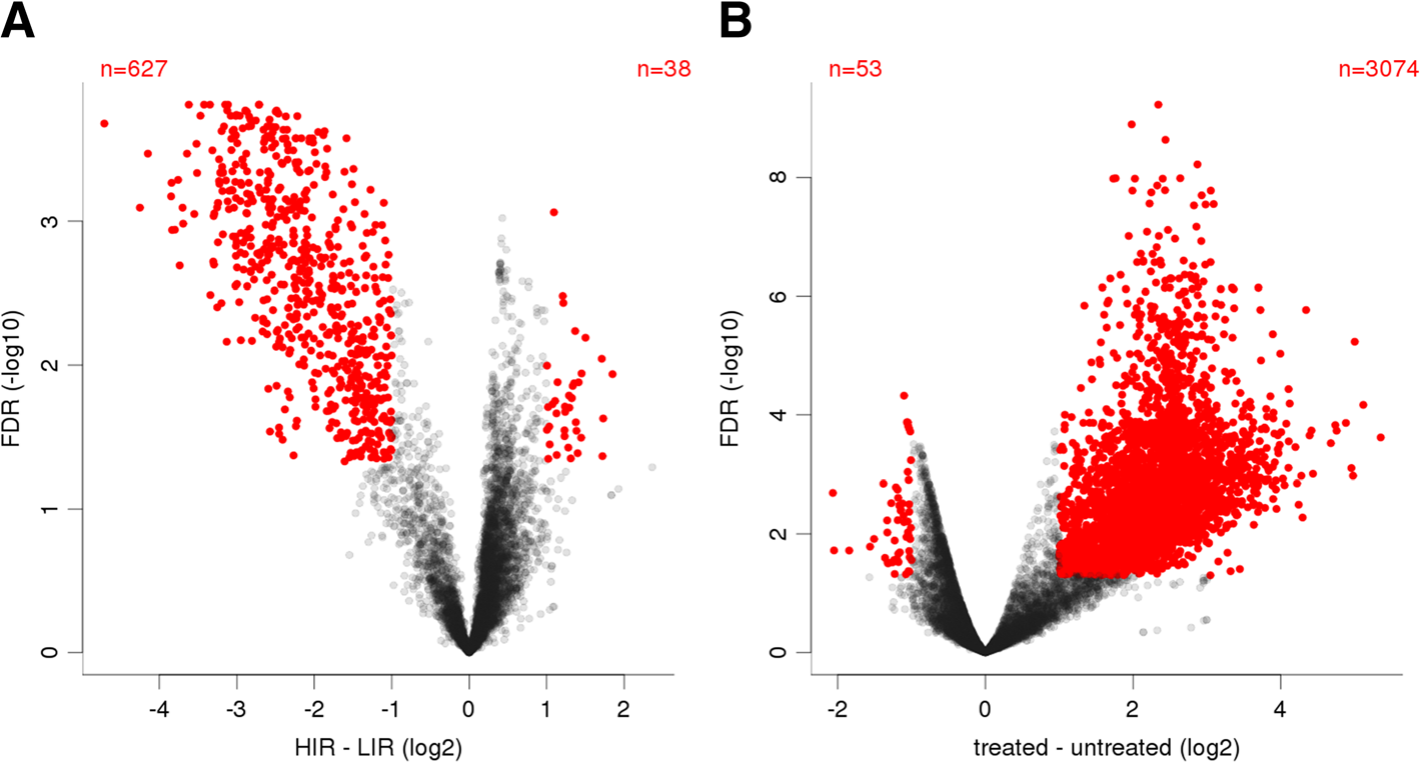
Volcano plots of lncRNA expression ratios: (**a**) Between the high (HIR) and low infertility risk (LIR) groups or (**b**) in the HIR group before and after GnRHa treatment. Genes with no significant difference in expression between the two groups compared in each panel are in black. Differentially expressed genes are shown in red. The most upregulated genes on the right, the most downregulated genes on the left, and the most significant genes at the top

Next, we compared samples obtained from HIR patients at the time of initial surgery (Fig. 2, lanes 5 and 6) and after six months of treatment with GnRHa (Fig. 2, lanes 7 and 8). The genes were ordered using an unsupervised clustering method (hierarchical clustering with complete linkage using Euclidean distances) and are shown in a false-color heatmap relative to the mean expression of each gene over all samples in Fig. 2. The results indicate that a large number of lncRNAs accumulate at low levels in the testes of boys with HIR compared to LIR, and that a substantial fraction of these transcripts is up-regulated by GnRHa treatment. In contrast, surgery alone had no significant impact on lncRNA expression. We explored the dataset using Volcano plots that display statistical significance (false discovery rate, FDR*)* against fold-change of expression signals allowing the selection of genes for which large and significant differences in expression levels were observed (Fig. 3).

We found that 627 and 38 lncRNAs were expressed at lower and higher levels, respectively, in HIR versus LIR samples (Fig. 3a). We concluded that the vast majority of differentially expressed lncRNAs are detected at lower levels in HIR testes. Comparing HIR testes before and after GnRHa treatment, we found that 3074 lncRNAs were increased, whereas 53 were decreased (Fig. 3b). Thus, hormonal treatment induces a considerable number of lncRNAs. In the following section the novel lncRNAs were organized based on their known functions or roles that were attributed to their potential target genes.

### Certain testicular lincRNAs upregulated by GnRHa treatment are involved in stem cell renewal, signaling, and cell differentiation

We also sought to gain insight into the potential roles that hormone-responsive RNAs might play by interpreting their genomic location, association with protein coding genes in sense/antisense pairs, and RNA-RNA interactions. We selected 11 of 77 lincRNAs and four of 46 AS-lncRNAs with > 2.0-fold change after GnRHa treatment because their expression patterns lead us to hypothesize that they are important for the development of Ad spermatogonia (Table 1). In this section we focus on novel potential regulatory lincRNAs and we provide context information about their putative protein-coding target genes. This includes previously published expression data obtained with samples from HIR and LIR patients (fold change and FDR values) [12] and functional information relevant for germ cells growth and differentiation from the literature.

**Table 1:** Testicular lincRNAs and AS-lncRNAs that increase after GnRHa treatment and are involved in stem cell renewal and differentiation

Gene ID	RPKM before GnRHa Median MAD	RPKM after GnRHa Median MAD	log2FC GnRH	*p*-value	FDR
*LINC-ROR*	0.044 / 0.06	0.40 / 0.24	2,68	0,0004	0,002
*LINC00261*	0.11 / 0.04	1.11 / 0.68	2.60	3.588E-08	4.35E-06
*LINC00293*	0.05 / 0.04	0.66 / 0.49	2.80	0.0001	0.001
*LINC00303*	0.22 / 0.07	1.25 / 0.45	2.57	0.0001	0.0008
*LINC00520*	0.15 / 0.07	0.79 / 0.64	2.76	0.0002	0.001
*LINC00898*	0.04 / 0.03	0.35 / 0.25	2.72	0.0002	0.001
*LINC00974*	0.07 / 0.04	0.48 / 0.46	3.9	0.0008	0.003
*LINC00994*	0.20 / 0.10	1.41 / 0.81	2.73	5.773E-06	0.0001
*LINC01016*	0.15 / 0.03	1.32 / 0.70	3,36	2.078E-09	6.43E-07
*LINC01121*	0.25 / 0.04	1.84 / 0.45	2.89	2.049E-09	6.43E-07
*LINC01553*	0.09 / 0.06	1.26 / 0.67	3.60	0.0002	0.001
*EGFR-AS1*	0.03 / 0.05	0.58 / 0.30	2.99	3.850E-07	2.28E-0.5
*HOTTIP*	0.04 / 0.03	0.47 / 0.37	2.22	0.0010	0.004
*MTOR-AS1*	0.21 / 0.19	1.90 / 1.33	4.96	6.67E-06	0.0001
*OTX2-AS1*	0.09 / 0.007	0.91 / 0.45	2.37	2.95E-0.6	8.83E-05

The log-fold changes (FC), p-value, false discovery rate (FDR), median expression values in reads per kilobase per million (RPKM) (Median), and the median absolute deviation (MAD) for LINC samples before and after GnRHa treatment are given

*LINC01016* is a so-called hub RNA that binds many mRNAs (encoding epigenetic regulators and transcription factors) and lincRNAs (including *XIST*). This feature distinguishes a hub RNA from most other transcripts that interact with few, one or no other RNAs. *LINC01016* is expressed in the same direction as *MLN* (Motilin) and is a transcriptional target of the estrogen receptor [15] (Table 1).

*LINC01121* is expressed upstream of *SIX2* and may influence its proximal promoter regions. *SIX2* interacts with *TCF7L2* and *OSR1* in a canonical WNT signaling-independent pathway, preventing the transcription of differentiation genes in cap mesenchyme, such as *WNT4* [16–18] (Table 1).

*LINC00261* is expressed in the 3’regions of *PAX1* and *FOXA2*, which encode transcription factors. It is also a hub lncRNA that binds mRNAs and lncRNAs, including *HOTAIR*, and is an epigenetically regulated tumor suppressor that is essential for activation of the DNA damage response [19]. FOXA2 is involved in androgen receptor regulation [19–21] and upregulated after GnRHa treatment (log2FC = 1.69; FDR = 0.0004;). Furthermore, *LINC00261* is a tumor suppressor that blocks cellular proliferation by activating the DNA damage response [22].

*LINC0030*3 is expressed upstream of *SOX13*, a developmental factor expressed in mouse Leydig cells and germ cells [23]. Therefore, this lncRNA could be involved in *SOX13* regulation. *LINC00293* is expressed upstream of *SPIDR*, which is involved in double-stranded break repair and genome integrity and binds two TTTY type testis-specific lncRNAs. Several lincRNAs involved in DNA damage repair were increased after GnRHa treatment, including *LINC00994* (expressed upstream of *PSMD6*), LINC00898 (binds mRNA encoding USP1), and testis-specific *LINC01553* (interacts with mRNA encoding *TIMELESS*). *TIMELESS* plays an important role in the control of DNA replication, the maintenance of genome stability throughout normal DNA replication, and regulation of the circadian clock [24]. (Table 1).

*EGFR-AS1,* which is involved in determining period length and in the DNA damage-dependent phase advancing the circadian clock [25], interacts with *NEU3* mRNA. *NEU3* activity enhances *EGFR* activation without affecting *EGFR* expression. This may indicate a regulatory mechanism involving a feedback loop. EGFR-AS1 is weakly expressed in adult testis and highly expressed in liver and liver cancer. Intense EGFR immunostaining was found in men with high plasma FSH levels and in all patients who received exogenous FSH, supporting a possible gonadotropin role in the modulation of EGFR expression [25]. GnRHa treatment increased the plasma FSH level and *EGFR-AS1*, but decreased *EGFR* expression (log2FC = − 0.58; FDR = 0.01;). Epidermal growth factor receptor signaling is associated with the pathogenesis of cutaneous squamous cell carcinoma. *LINC00520*-targeted *EGFR* inhibition might result in inactivation of the PI3K/Akt pathway, thereby inhibiting cancer development [26].

*HOTTIP* mediates the regulation of *CXCL* genes, which are implicated in Ad spermatogonia differentiation [12]. *HOTTIP* is antisense to *HOXA13* and modulates cancer stem cell properties in human pancreatic cancer by regulating *HOXA9* [27, 28]. *OTX2-AS1* is a NAT RNA that plays an important role in eye development and exhibits sequence complementarity to the exon sequences in its corresponding sense gene, *OTX2*, in both mice and humans [12]. *OTX2* is downregulated in HIR (log2FC = − 1.73; FDR = 0.02;) and upregulated after GnRH treatment (log2FC = 1.24; FDR = 0.03) [12]. Though no role has been found for *OTX2-AS1*, deletion of its sense gene *OTX1* was found in six patients with genitourinary defects. Three of these individuals were diagnosed with cryptorchidism [29]. *MTOR*, the key regulator of spermatogenesis [30], is downregulated in boys with HIR (log2FC = − 0.42; FDR = 0.03;) and remains downregulated after GnRHa treatment (log2FC = − 0.53; FDR = 0.02;). Its antisense gene, *MTOR-AS1*, was up-regulated 4.9-log2 by GnRHa treatment (Table 1). Thus far, nothing is known about the function of *MTOR-AS1*.

*LINC-ROR* is induced 6.5-fold by GnRHa. (Table 1) This lncRNA controls stem cell renewal and acts as an miRNA sponge via gene silencing, which indicates that the transcript itself has a biological role [31–33]. In addition, we found that *BOD1L2*, a testis-specific gene, is located downstream of *LINC-ROR* and may be transcriptionally regulated by the lncRNA. *BOD1L2* plays a role in chromosome biorientation through the detection or correction of syntelic attachments in mitotic spindles [34, 35]. Buserelin treatment increases *BOD1L2* gene expression (log2FC = 1.72 l; FDR = 0.003;), indicating a possible role for it in Ad spermatogonia differentiation.

### LincRNAs downregulated in HIR testes and stimulated after GnRHa treatment are associated with cancer and the transition of ad spermatogonia

We previously reported different lncRNA expression in patients with HIR compared to LIR; some of these RNAs participate in epigenetic processes, including *AIRN, ERICH-AS1, FENDRR, HAGLR*, and *XIST* [12]. Here, we focuse on seven lncRNAs with decreased gene expression in HIR, indicating abrogated mini-puberty, and increased expression after GnRHa treatment (Tables 2 and 3).

**Table 2:** Testicular lincRNAs downregulated in HIR testes compared to LIR

lincRNA	LIR Median/MAD	HIR Median/ MAD	log2FC	*p*-value	FDR
*LINC00922*	0.47 0.17	0.10 0.07	- 1.47	0.002	0.01
*LINC00221*	0.64 0.27	0.27 0.07	- 1.20	0.0007	0.008
*LINC01249*	0.43 0.28	0.15 0.08	- 1.42	0.001	0.01
*LINC00701*	0.29 0.03	0.11 0.06	- 0.84	0.002	0.02
*HOTAIR*	0.49 0.23	0.13 0.09	- 1.74	0.0001	0.002
*DLX6-AS1*	0.42 0.09	0.20 0.06	- 0.92	0.006	0.03
*LINC01446*	0.57 0.09	0.31 0.08	- 1.21	0.0002	0.003

The log-fold changes (FC), *p*-value, false discovery rate (FDR), median expression values in reads per kilobase per million (RPKM) (Median), and the median absolute deviation (MAD) for LINC samples are presented

**Table 3:** LncNRAs downregulated in HIR testes and stimulated after GnRHa treatment

lincRNA (RPKM)	before GnRHa Median MAD	after GnRHa Median MAD	log2FC	p-value	FDR
*LINC00922*	0.10 0.07	0.85 0.40	1.41	0.02	0.04
*LINC00221*	0.27 0.07	1.12 0.42	1.23	0.01	0.03
*LINC01249*	0.15 0.08	0.92 0.56	1.38	0.003	0.01
*LINC00701*	0.11 0.06	0.72 0.48	1.28	0.0009	0.003
*HOTAIR*	0.13 0.09	1.04 0.87	1.14	0.01	0.03
*DLX6-AS1*	0.20 0.06	1.13 0.81	2.02	1.55E-05	0.0002
*LINC01446*	0.31 0.08	1.49 0.82	1.14	0.0007	0.003

The log-fold changes (FC), p-value, false discovery rate (FDR), median expression values in reads per kilobase per million (RPKM) (Median), and the median absolute deviation (MAD) for LINC samples before and after GnRHa treatment are shown

*LINC00922* is expressed upstream of cadherins *CDH5* and *CDH11*; the latter encodes a calcium-dependent cell adhesion protein that may play a role in testicular architecture [36].

*LINC0022*1 binds mRNA encoding DCBLD2, which is involved in negative regulation of cell growth. Significant downregulation of *DCBLD2* occurred after GnRHa treatment (log2FC = − 1.0; FRD = 0.0002;). *LINC00221* interacts with *VPS53*’s mRNA. The protein acts as component of the GARP complex involved in retrograde transport from early and late endosomes to the trans-Golgi network [37].

*LINC01249* is expressed upstream of *SOX11*, which is important for embryonic neurogenesis and tissue modeling. *SOX11* is upregulated after GnRHa treatment (log2FC = 0.7; FDR = 0.008). It has been suggested that, together with *SOX4*, *SOX11* may function as a transcriptional repressor in fetal testes, contributing to the precise regulation of *SRY* and *SOX9* [23].

*LINC01446* promotes glioblastoma progression by modulating the miR-489-3p/*TPT1* pathway [38].

The testis expression of *LINC00701* is developmental stage-specific and associated with *SLC25A37*, encoding a solute carrier localized in the inner mitochondrial membrane. The protein functions as an essential iron importer for the synthesis of mitochondrial heme and iron-sulfur clusters [39].

*HOX* antisense intergenic RNA *(HOTAIR)* is an lncRNA that coordinates with chromatin-modifying enzymes, regulates gene silencing, and is transcriptionally induced by estradiol (E2) [12, 40, 41].

Distal-less homeobox6 antisense (*DLX6-AS1*) was downregulated in HIR and responded positively to GnRHa treatment. This supports the observation in mice that *DLX6* participates in the control of steroidogenesis [42]. *DLX5* and *DLX6* showed low or no expression in HIR samples.

*TINCR,* an lncRNA required for the induction of key differentiation genes, is downregulated in HIR testes (log2FC = − 1.07 l; FDR = 0.002). Seven epigenetic modifiers found to bind *TINCR* were upregulated in HIR and downregulated after GnRHa treatment (Table 4).

**Table 4:** Seven epigenetic modifiers that bind TINCR

Gene ID	Name	Log2FC HIR/LIR	FDR HIR/LIR	Log2FC HIR/ GnRHa	FDR HIR/ GnRHa
*SETD7*	SET domain containing lysine methyltransferase 7	+ 0.22	0.042	−0.85	0.0006
*ARID4B*	AT-rich ineraction domain 4B	+ 0.18	0.041	−0.63	0.008
*ARID5B*	AT-rich interaction domain 5B	+ 0.35	0.004	−0.49	0.003
*KDM6A*	lysine demethylase 6A	+ 0.20	0.017	−0.81	0.0009
*CHD6*	chromodomain helicase DNA binding protein 6	+ 0.21	0.040	−0.54	0.03
*MBD2*	methyl-CpG binding domain protein 2	+ 0.23	0.029	−0.68	0.004
*BPTF*	bromodomain PHD finger transcription factor	+ 0.19	0.055	−0.61	0.01

The log-fold changes (FC), p-value, false discovery rate (FDR), comparing HIR and LIR cryptorchid testes as well as results from HIR group following GnRHa treatment are presented

## Discussion

In this study, we aimed to gain molecular insight into the effect on testicular lncRNA expression levels of a curative treatment for cryptorchidism and related infertility that combines surgery and nasal administration of GnRHa [11, 12, 14]. We found hundreds of lncRNAs that respond to treatment, including a subset that is present at lower levels in testicular samples from boys with HIR. A detailed interpretation of the expression data revealed candidate lncRNAs that may play important regulatory roles in establishing adult spermatogenesis during early postnatal development in humans. Our data are consistent with the hypothesis that hypogonadotropic hypogonadism in boys with altered mini-puberty is the consequence of a profoundly altered gene expression program involving protein-coding genes and lncRNAs. The results point to molecular mechanisms that underlie the ability of GnRHa to rescue fertility.

### Study design for human testicular RNA profiling experiments

When working with human samples, a critical issue is the number of cases included in a given analysis. The number of replicates affects the statistical confidence level, and human tissue samples exhibit intrinsic variability that needs to be controlled. In this exploratory lncRNA profiling study, we included first seven patients chosen sequentially from a study based on randomized patient samples [12, 14]. Their inclusion in the cohorts to be treated or to remain untreated was completely unbiased by any parameter other than undescended testes, which were surgically corrected. This sample size, while small, is enough for an initial transcriptome study as presented here.

### Curative hormone treatment affects signaling pathways

During GnRHa treatment, increased LH and testosterone secretion induced the transition of gonocytes and undifferentiated spermatogonia into Ad spermatogonia. In this context, it is interesting that the expression of *LINC-ROR*, a key regulator of pluripotent stem cell reprogramming, increased after hormone treatment. LINC-ROR influences cell differentiation, in part, by acting as a sponge for miR-138 and miR-145 and by activating both the canonical and non-canonical WNT/β-catenin signaling pathways [31]. Importantly, an increase in *mTOR-AS1* expression after GnRHa treatment may have resulted in the suppression of *mTOR* activity, allowing spermatogonial stem cells to undergo self-renewal. *WNT3* induces many transcription factors associated with mesoderm and is downregulated in the testes of men with HIR testes [12]. *WNT interacts with mTOR* signaling to affect cancer cell growth and tumor metabolism [43], as well as the formation of spermatozoa [30]. We propose that *WNT3* is the signaling component that regulates early expression of *Brachyury (T)* [44], a mesodermal factor known to determine the fate of Ad spermatogonia. T is a classical and conserved mesodermal factor essential for robust activation of the germline determinants *PRDM1* and *PRDM14* [45]. T directly upregulates these genes, thereby delineating the downstream primordial germ cell program. In mutant mice lacking *Bmp4*, a program induced by *WNT3* prevents T from activating *PRDM1* and *PRDM14*, demonstrating a permissive role of *Bmp4* in primordial gem cell specification [45]. We found that *DMRTC2*, *PAX7*, *T*, and *TERT* are downregulated in testes with defective mini-puberty and respond to GnRHa treatment [46]. Furthermore, we found lower levels of *PRDM1*, *PRDM6*, *PRDM9*, *PRDM13*, and *PRDM14* mRNA in the testes of patients with HIR compared to LIR, and *PRDM7*, *PRDM9*, *PRDM12*, and *PRDM16* were significantly induced after GnRHa treatment. Thus, GnRHa treatment induces an alternate pathway to stimulate *PRDM9* for Ad spermatogonia specification without the permissive role of *BMP4*, but increased the expression of *BMP5* (log2FC = 2.31; FDR = 0.0001;) [12, 47]. *LINC01121* is expressed upstream of *SIX2* and may therefore influence its expression. *SIX2* and *WNT* regulate the self-renewal and commitment of nephron progenitors through shared gene regulatory networks [16–18]. *SIX2* also activates the expression of *GDNF* and plays a role in cell proliferation and migration. Testes in men with HIR expressed lower levels of *SIX2* than LIR testes (log2FC = − 1.76; FDR = 0.0008). GnRHa treatment increased *SIX2* (log2FC = 1.61; FDR = 0.014) and *GDNF* (log2FC = 1.46; FDR = 0.003) expression. Taken together, our results support the notion that *LINC-ROR* and *mTOR* are involved in Ad spermatogonia development and specification via the WNT signaling pathway.

### Hormone treatment influences epigenetic factors

*LINC00261* stimulates the expression of *HOTAIR* and *HOTTIP* genes to stabilize androgen receptor (AR) together with *FOXA1*. Specifically, the DNA-binding domain (DBD)/hinge region of AR directly interacts with the fork head domain of *FOXA1*, thereby acting as an AR-collaborating factor [48].

*HOTAIR’s* promoter contains multiple functional estrogen response elements (EREs). *HOTAIR* mediates the recruitment of H3K27 methyltransferase and H3K4 demethylase, which leads to efficient repression of certain loci. The levels of histone H3 lysine-4 trimethylation, histone acetylation, and RNA polymerase II recruitment are increased at the *HOTAIR* promoter in the presence of E2, and knock-down of ERs downregulated E2-induced *HOTAIR* expression. Thus, like the transcription of protein-coding genes, E2 induces the transcription of antisense RNAs [27, 49, 50]. HIR patient’s exhibit decreased *HOTAIR* levels, [12] and GnRHa treatment induced *HOTAIR* expression, indicating that LH, testosterone and/or converted E2 have a positive effect on *HOTAIR* and, thus, Ad spermatogonia differentiation [12]. Furthermore, *HOTAIR* was previously shown to mediate tumorigenesis by recruiting *EZH2* [51]. This is of interest, as GnRHa induces expression of *HOTAIR*, downregulates *EZH2* (log2FC = − 0.6; FDR = 0.01) and implicates the regulation of *HOTTIP* gene transcription, which then transcriptionally regulates the HOXA cluster. As a result, an increase occurs in *HOXA2* (log2FC = 2.38; FDR = 0.007), *HOXA3* (log2FC = 1.68; FDR = 0.007), *HOXA11* (log2FC = 1.77; FDR = 0.02), and HOXA-AS3 (log2FC = 2.68; FDR = 0.0001).

Gamma-aminobutyric acid (GABA) plays a key developmental role in the regulation of GnRH neuron migration from the olfactory placodes into the forebrain during fetal development [52], and co-expression of *DLX3* and *PAX6* proteins, correlates with acquisition of the olfactory placode fate [53]. Moreover, *GABA-A* receptors and GABA transporter 1 (*GAT1*) have been reported to be involved in the proliferation of Leydig cells, testosterone production, and spermatogenesis [54]. GABRA 3 (log2FC = − 2.49; FDR = 0.0001;) and GABR5 (log2FC = − 2.59; FDR = 0.0006;) were downregulated in HIR testes. Following GnRHa treatment, an increase expression was observed in *DLX3*, *PAX6* [12], and *TP63* (log2FC = 0.91; FDR = 0.002;), whereby the latter was downregulated in HIR (log2FC = − 1.58; FDR = 0.001;). Berghoff et al. proposed a model in which DLX6-AS1 inhibits the ultraconserved DNA methylation mark in *DLX5*/*6*, facilitating antagonistic interactions between repressive and activating transcription factors *MECP2* and *DLX* [53]. *DLX2, DLX3, DLX5* and *DLX6* showed low or no expression in HIR samples [12] Loss of *DLX1*/*2* increases site-specific methylation of the *DLX5*/*6* ultraconserved enhancer [53]. Tp63 regulates *DLX5* and *DLX6* transcription, at least in part, via cis-acting regulation at the promoter level [55]. These interactions allow differential control of adjacent genes by shared DNA regulatory elements [56]. It was also shown that deletion of *Dlx5* and *Dlx6* in the mouse leads to decreased testosterone levels and an abnormal masculinization phenotype [42]. Thus, impaired steroidogenesis during mini-puberty in HIR boys may be induced by altered GABRA-A receptor signaling, silenced *DLX6* expression as well as destabilization of AR and ERs.

### GnRHa treatment affects genes associated with normal and pathological cell division

Several recent studies have reported roles for lncRNAs in cancer, including *MALAT1* and *GAS,* strongly indicating that lncRNAs not only control gene regulatory pathways in normal cells and tissue, but also during tumor development [57, 58]. The occurrence and progression of cancer is the result of a combination of multiple factors. Furthermore, cancer-specific lncRNA expression patterns appear to be more tissue- and stage-specific than those of protein-coding genes, supporting the potential development of lncRNAs as efficient biomarkers and therapeutic targets [58]. Interestingly, GnRHa treatment increased LH and testosterone secretion and *HOTAIR* expression, and probably its recruitment to chromatin, whereas the expression of *MALAT1* was reduced (log2FC = − 0.64; FDR = 0.03; REF), suggesting opposite regulation and functions of these lncRNAs during normal testis development. It was reported that LINC-ROR promote liver cancer stem cell growth by upregulating *TERT* and *C-MYC* [59]. Notably, GnRHa treatment stimulates the expression of *TERT* (log2FC = 0.91; FDR = 0.002;) [46], which is decreased in HIR (log2FC = − 1.58; FDR = 0.001;) [46] but downregulates *C-MYC* signaling (log2FC = − 0.58; FDR = 0.01;).

Variation in lincRNA expression may be associated with cancer progression. For example, *LINC00261*, which plays an important role in gastric cancer, is stimulated by GnRHa, and exerts tumor suppressive activity by reducing cancer cell invasion via suppression of the epithelial-mesenchymal transition process [60]. Another lincRNA, *TINCR,* is involved in normal tissue differentiation and plays a critical role in cancer and metastasis. *TINCR* expression is downregulated in HIR (log2FC = − 1.07; FDR = 0.002;) [12]. We observed that *TINCR* depletion in HIR testes resulted in the induction of key epigenetic modifiers, seven of which were downregulated following GnRHa treatment (Table 4). One of them, *SETD7*, is an epigenetic modifier and regulates AR [61]. *SETD7* binds *TINCR* and may mediate the formation of AR-associated coactivator complexes. Taken together, the results indicate that the HIR group of cryptorchid boys with abortive mini-puberty expresses several cancer genes. Gonadotropin-releasing hormone treatment may protect against testicular tumor development by downregulating oncogenes, such as *MALAT1, mTOR*, C-MYC, and *EZH2* to enable normal germ cell development.

## Conclusions

Two major goals in the field of male reproductive biology are to elucidate the molecular mechanisms that underlie cryptorchidism and to develop an effective treatment for its long-term consequences. According to the mini-puberty hypothesis, early-life exposure to gonadal hormones during a specific window of sensitivity triggers sex-specific developmental processes. To preserve molecular features of differentiated cells, it is crucial that transcriptional alterations triggered by external or intrinsic signals continue beyond the initial stimulus. One possible mechanism involves epigenetic histone variant replacement, chromatin remodeling factors, and lncRNAs associated with epigenetic factors. We found that many of the lincRNAs responding to GnRHa treatment are associated with somatic cancer. This may reflect the fact that the hormone stimulates germ stem cell growth and Leydig, as well as Sertoli, cell division. The mechanisms involved are likely rather diverse and may include promoter/enhancer activity, miRNA sponge activity, or control of gene expression via RNA-RNA interactions. We propose that the lncRNAs identified in this study may be involved in establishing normal male fertility by acting at early stages of spermatogonia stem cell development and by affecting other testicular cells capable of responding to GnRHa treatment. Our results provide information’s for further functional analysis of long-noncoding RNA in relation to the infertility development. We propose that the HOTAIR and DLX pathways, as well as both canonical and non-canonical WNT pathways, are involved in Ad spermatogonia growth and differentiation.

## Data Availability

Raw data files were deposited at the Database of Genotypes and Phenotypes (dbGaP) under accession number phs001275.v1.p1.

## Abbreviations

1PRDM6: PR/SET domain 6
AIRN: Antisense of IGF2R (insulin like growth factor 2 receptor) non-protein coding RNA
AR: Androgen receptor
ARID4B: AT-rich interaction domain 4B
ARID5B: AT-rich interaction domain 5B
AS-lncRNA: Antisense long non-coding RNA
Bmp4: Bone morphogenetic protein 4
BMP5: Bone morphogenetic protein 5
BOD1L2: Biorientation of chromosomes in cell division 1 like 2
BPTF: Bromodomain PHD finger transcription factor
CDH11: Cadherin 11
CDH5: Cadherin 5
CDH6: Cadherin 6
C-MYC: v-myc avian myelocytomatosis viral oncogene homolog
CXCL: Chemokine
DBD: DNA-binding domain
dbGaP: Database of Genotypes and Phenotypes
DCBLD2: Discoidin, CUB and LCCL domain containing 2
DLX: Distal-less homeobox
DLX2: Distal-less homeobox 2 [*Homo sapiens* (human)]
DLX3: Distal-less homeobox 3 [*Homo sapiens* (human)]
*Dlx5*: Distal-less homeobox 5 [*Mus musculus* (house mouse)]
*Dlx6*: Distal-less homeobox 6 [*Mus musculus* (house mouse)]
DLX6-AS1: Distal-less homeobox 6 antisense RNA 1
DMRTC2: DMRT (doublesex and mab-3 related transcription factor) like family C2
E2: Estradiol
EGFR: Epidermal growth factor receptor
EGFR-AS1: Epidermal growth factor receptor antisense RNA 1
EREs: Estrogen response elements
ERICH-AS1: Glutamate rich protein antisense RNA 1
ERs: Estrogen receptors
ESC: Embryonic stem-cell
EZH2: Enhancer of zeste 2 polycomb repressive complex 2 subunit
FDR: False discovery rate
FENDRR: FOXF1 (forkhead box F1) adjacent non-coding developmental regulatory RNA
FOXA1: Forkhead box A1
FOXA2: Forkhead box A2
FSH: Follicle stimulating hormone
GABA: Gamma-aminobutyric acid
GABA-A: Gamma-aminobutyric acid type A
GABRA-A: gamma-aminobutyric acid (GABA) A receptor, alpha 1
GABRA-A5: Gamma-aminobutyric acid (GABRA) A receptor 5
GARP: Golgi-associated retrograde protein
GAS: Growth arrest specific
GAT1: GABA (gamma-aminobutyric acid) transporter 1
GDNF: Glial cell derived neurotrophic factor
GnRHa: Gonadotropin releasing hormone antagonist
H3K27: Histone H3 Lysine 27
H3K4: Histone H3 Lysine 4
HAGLR: HOXD (homeobox D cluster) antisense growth-associated long non-coding RNA
HIR: High infertility risk
HOTAIR: HOX (homeobox) transcript antisense RNA
HOTTIP: HOXA (homeobox A cluster) distal transcript antisense RNA
HOXA11: Homeobox A11
HOXA13: Homeobox A13
HOXA2: Homeobox A2
HOXA3: Homeobox A3
HOXA9: Homeobox A9
HOXA-AS3: HOXA (Homeobox A) cluster antisense RNA 3
KDM6A: Lysine demethylase 6A
LH: Luteinizing hormone
LINC00221: Long intergenic non-protein coding RNA 221
LINC00261: Long intergenic non-protein coding RNA 261
LINC00282: long intergenic non-protein coding RNA 282
LINC00293: Long intergenic non-protein coding RNA 293
LINC00303: Long intergenic non-protein coding RNA 303
LINC00520: Long intergenic non-protein coding RNA 520
LINC00701: Long intergenic non-protein coding RNA 701
LINC00898: Long intergenic non-protein coding RNA 898
LINC00922: Long intergenic non-protein coding RNA 922
LINC00974: Long intergenic non-protein coding RNA 974
LINC00994: Long intergenic non-protein coding RNA 994
LINC01016: Long intergenic non-protein coding RNA 1016
LINC01121: Long intergenic non-protein coding RNA 1121
LINC01249: Long intergenic non-protein coding RNA 1249
LINC01446: Long intergenic non-protein coding RNA 1446
LINC01553: Long intergenic non-protein coding RNA 1553
lincRNA: Long intergenic non-protein coding RNA
Linc-ROR: Long intergenic non-protein coding RNA, regulator of reprogramming
LIR: Low infertility risk
lncRNA: Long non-coding RNA
log2FC: log2 fold change
MALAT1: Metastasis associated lung adenocarcinoma transcript 1
MBD2: Methyl-CpG binding domain protein 2
MECP2: methyl-CpG binding protein 2
miR-138: microRNA 138
miR-145: microRNA 1
miR-489-3P: microRNA 489
MLN: Motilin
MTOR: mechanistic target of rapamycin kinase
MTOR-AS1: MTOR (mechanistic target of rapamycin kinase) antisense RNA 1
NAT RNA: N-acetyltransferase RNA
NATs: Natural antisense transcripts
NEU3: Neuraminidase 3
OSR1: Odd-skipped related transcription factor 1
OTX1: Orthodenticle homeobox 1
OTX2: Orthodenticle homeobox 2
OTX2-AS1: Orthodenticle homeobox 2 antisense RNA 1 (head to head)
PAX1: Paired box 1
PAX6: Paired box 6
PAX7: Paired box 7
PI3K/Akt pathway: Phosphatidylinositol 3-kinase, putative, and protein kinase B pathway
PRDM1: PR domain containing 1, with ZNF domain
PRDM12: PR/SET domain 12
PRDM14: PR/SET domain 14
PRDM16: PR/SET domain 16
PRDM7: PR/SET domain 7
PRDM9: PR/SET domain 9
PRMD13: PR/SET domain 13
PSMD6: Proteasome 26S subunit, non-ATPase (adenosine triphosphatase) 6
RPKM: Reads per kilo base per million mapped reads
S/T: Total germ cell count per tubule
SETD7: SET domain containing 7, histone lysine methyltransferase
SIX2: SIX homeobox 2
SLC25A37: Solute carrier family 25 member 37
SOX11: SRY (sex determining region Y)-box transcription factor 11
SOX13: SRY (sex determining region Y)-box transcription factor 13
SOX4: SRY (sex determining region Y)-box transcription factor 4
SPIDR: Scaffold protein involved in DNA repair
SRY: Sex determining region of Y
T: Brachyury
TCF7L2: Transcription factor 7 like 2
TERT: Telomerase reverse transcriptase
TIMELESS: Timeless circadian regulator
TINCR: TINCR (Tissue differentiation-inducing non-protein coding RNA) ubiquitin domain containing
TP63: Tumor protein p63
TPT1: Tumor protein, translationally-controlled 1
TTTY: Testis-specific transcript, Y-linked
USP1: Ubiquitin specific peptidase 1
VPS53: VPS53 (Vacuolar protein sorting 53 homolog) subunit of GARP (Golgi-associated retrograde protein) complex
Wnt: Wingless-related integration site
WNT3: Wnt family member 3
WNT4: Wnt family member 4
XIST: Inactive X specific transcripts

## References

[1] Wang KC, Chang HY (2011). Molecular mechanisms of long noncoding RNAs. Mol Cell.

[2] Pelechano V, Steinmetz LM (2013). Gene regulation by antisense transcription. Nat Rev Genet.

[3] Tufarelli C, Stanley JA, Garrick D, Sharpe JA, Ayyub H, Wood WG (2003). Transcription of antisense RNA leading to gene silencing and methylation as a novel cause of human genetic disease. Nat Genet.

[4] Fernandes JCR, Acuña SM, Aoki JI, Floeter-Winter LM, Muxel SM (2019). Long non-coding RNAs in the regulation of gene expression: physiology and disease. Noncoding RNA.

[5] Hadjicharalambous MR, Lindsay MA (2019). Long non-coding RNAs and the innate immune response. Noncoding RNA.

[6] Carninci P, Kasukawa T, Katayama S, Gough J, Frith MC, Maeda N (2005). The transcriptional landscape of the mammalian genome. Science..

[7] Djebali S, Davis CA, Merkel A, Dobin A, Lassmann T, Mortazavi A (2012). Landscape of transcription in human cells. Nature..

[8] Forrest AR, Kawaji H, Rehli M, Baillie JK, de Hoon MJ, Haberle V (2014). FANTOM consortium and the RIKEN PMI and CLST (DGT): a promoter-level mammalian expression atlas. Nature..

[9] Hadziselimovic F, Herzog B (2001). The importance of both an early orchidopexy and germ cell maturation for fertility. Lancet..

[10] Hadziselimovic F, Hocht B, Herzog B, Buser MW (2007). Infertility in cryptorchidism is linked to the stage of germ cell development at orchidopexy. Horm Res.

[11] Hadziselimovic F (2008). Successful treatment of unilateral cryptorchid boys risking infertility with LH-RH analogue. Int Braz J Urol.

[12] Hadziselimovic F, Gegenschatz-Schmid K, Verkauskas G, Demougin P, Bilius V, Dasevicius D (2017). GnRHa treatment of Cryptorchid boys affects genes involved in hormonal control of the HPG Axis and fertility. Sex Dev.

[13] Verkauskas G, Malcius D, Eidukaite A, Vilimas J, Dasevicius D, Bilius V (2016). Prospective study of histological and endocrine parameters of gonadal function in boys with cryptorchidism. J Pediatr Urol.

[14] Vincel Beata, Verkauskas Gilvydas, Bilius Vytautas, Dasevicius Darius, Malcius Dalius, Jones Birute, Hadziselimovic Faruk (2018). Gonadotropin-Releasing Hormone Agonist Corrects Defective Mini-Puberty in Boys with Cryptorchidism: A Prospective Randomized Study. BioMed Research International.

[15] Jonsson P, Coarfa C, Mesmar F, Raz T, Rajapakshe K, Thompson JF (2015). Single-molecule sequencing reveals estrogen-regulated clinically relevant lncRNAs in breast Cancer. Mol Endocrinol.

[16] Park JS, Ma W, O'Brien LL, Chung E, Guo JJ, Cheng JG (2012). Six2 and Wnt regulate self-renewal and commitment of nephron progenitors through shared gene regulatory networks. Dev Cell.

[17] Liu Hongbing, Chen Shaowei, Yao Xiao, Li Yuwen, Chen Chao-Hui, Liu Jiao, Saifudeen Zubaida, El-Dahr Samir S. (2018). Histone deacetylases 1 and 2 regulate the transcriptional programs of nephron progenitors and renal vesicles. Development.

[18] Xu Xu J, Liu H, Park JS, Lan Y, Jiang R (2014). Osr1 acts downstream of and interacts synergistically with Six2 to maintain nephron progenitor cells during kidney organogenesis. Development..

[19] Fang Q, Sang L, Du S (2018). Long noncoding RNA LINC00261 regulates endometrial carcinoma progression by modulating miRNA/FOXO1 expression. Cell Biochem Funct.

[20] Liu Z, Dai J, Shen H (2018). Systematic analysis reveals long noncoding RNAs regulating neighboring transcription factors in human cancers. Biochim Biophys Acta Mol basis Dis.

[21] Connelly ZM, Yang S, Chen F, Yeh Y, Khater N, Jin R (2018). Foxa2 activates the transcription of androgen receptor target genes in castrate resistant prostatic tumors. Am J Clin Exp Urol.

[22] Shahabi S, Kumaran V, Castillo J, Cong Z, Nandagopal G, Mullen DJ (2019). LINC00261 is an epigenetically regulated tumor suppressor essential for activation of the DNA damage response. Cancer Res.

[23] Daigle M, Roumaud P, Martin LJ (2015). Expressions of Sox9, Sox5, and Sox13 transcription factors in mice testis during postnatal development. Mol Cell Biochem.

[24] Shen X, Li M, Mao Z, Yu W (2018). Loss of circadian protein TIMELESS accelerates the progression of cellular senescence. Biochem Biophys Res Commun.

[25] Foresta C, Varotto A (1994). Immunocytochemical localization of epidermal growth factor receptors in human testis from infertile subjects. Fertil Steril.

[26] Mei XL, Zhong S (2019). Long noncoding RNA LINC00520 prevents the progression of cutaneous squamous cell carcinoma through the inactivation of the PI3K/Akt signaling pathway by downregulating EGFR. Chin Med J.

[27] Lin C, Wang Y, Wang Y, Zhang S, Yu L, Guo C (2017). Transcriptional and posttranscriptional regulation of HOXA13 by lncRNA HOTTIP facilitates tumorigenesis and metastasis in esophageal squamous carcinoma cells. Oncogene..

[28] Cheng Y, Jutooru I, Chadalapaka G, Corton JC, Safe S (2015). The long noncoding RNA HOTTIP enhances pancreatic cancer cell proliferation, survival and migration. Oncotarget.

[29] Jorgez Carolina J., Rosenfeld Jill A., Wilken Nathan R., Vangapandu Hima V., Sahin Aysegul, Pham Dung, Carvalho Claudia M. B., Bandholz Anne, Miller Amanda, Weaver David D., Burton Barbara, Babu Deepti, Bamforth John S., Wilks Timothy, Flynn Daniel P., Roeder Elizabeth, Patel Ankita, Cheung Sau W., Lupski James R., Lamb Dolores J. (2014). Genitourinary Defects Associated with Genomic Deletions in 2p15 Encompassing OTX1. PLoS ONE.

[30] Xu H, Shen L, Chen X, Ding Y, He J, Zhu J (2016). mTOR/P70S6K promotes spermatogonia proliferation and spermatogenesis in Sprague Dawley rats. Reprod BioMed Online.

[31] Loewer S, Cabili MN, Guttman M, Loh YH, Thomas K, Park IH (2010). Large intergenic non-coding RNA-RoR modulates reprogramming of human induced pluripotent stem cells. Nat Genet.

[32] Wang Y, Xu Z, Jiang J, Xu C, Kang J, Xiao L (2013). Endogenous miRNA sponge lincRNA-RoR regulates Oct4, Nanog, and Sox2 in human embryonic stem. Dev Cell.

[33] Zou G, Liu T, Guo L, Huang Y, Feng Y, Huang Q (2016). miR-145 modulates lncRNA-ROR and Sox2 expression to maintain human amniotic epithelial stem cell pluripotency and β islet-like cell differentiation efficiency. Gene..

[34] Porter IM, McClelland SE, Khoudoli GA, Hunter CJ, Andersen JS, McAinsh AD (2007). Bod1, a novel kinetochore protein required for chromosome biorientation. J Cell Biol.

[35] Cleveland DW, Mao Y, Sullivan KF (2003). Centromere and kinetochores: from epigenetics to mitotic checkpoint signaling. Cell..

[36] Hawi Z, Tong J, Dark C, Yates H, Johnson B, Bellgrove MA (2018). The role of cadherin genes in five major psychiatric disorders: a literature update. Am J Med Genet.

[37] Liewen H, Meinhold-Heerlein I, Oliveira V, Schwarzenbacher R, Luo G, Wadle A (2005). Characterization of the human GARP (Golgi associated retrograde protein) complex. Exp Cell Res.

[38] Zhang L, Wang Q, Wang F, Zhang X, Zhang L, Tang Y (2018). LncRNA LINC01446 promotes glioblastoma progression by modulating miR-489-3p/TPT1 axis. Biochem Biophys Res Commun.

[39] Chen W, Paradkar PN, Li L, Pierce EL, Langer NB, Takahashi-Makise N (2009). Abcb10 physically interacts with mitoferrin-1 (Slc25a37) to enhance its stability and function in the erythroid mitochondria. Proc Natl Acad Sci U S A.

[40] Bhan A, Mandal SS (2015). LncRNA HOTAIR: a master regulator of chromatin dynamics and cancer. Biochim Biophys Acta.

[41] Bhan A, Mandal SS (2016). Estradiol-induced transcriptional regulation of long non-coding RNA, HOTAIR. Methods Mol Biol.

[42] Nishida H, Miyagawa S, Vieux-Rochas M, Morini M, Ogino Y, Suzuki K (2008). Positive regulation of steroidogenic acute regulatory protein gene expression through the interaction between Dlx and GATA-4 for testicular steroidogenesis. Endocrinology..

[43] Zeng H, Lu B, Zamponi R, Yang Z, Wetzel K, Loureiro J (2018). mTORC1 signaling suppresses Wnt/β-catenin signaling through DVL-dependent regulation of Wnt receptor FZD level. Proc Natl Acad Sci U S A.

[44] Di Ruscio A, Ebralidze AK, Benoukraf T, Amabile G, Goff LA, Terragni J (2013). DNMT1-interacting RNAs block gene-specific DNA methylation. Nature..

[45] Aramaki S, Hayashi K, Kurimoto K, Ohta H, Yabuta Y, Iwanari H (2013). A mesodermal factor, T, specifies mouse germ cell fate by directly activating germline determinants. Dev Cell.

[46] Gegenschatz-Schmid Katharina, Verkauskas Gilvydas, Demougin Philippe, Bilius Vytautas, Dasevicius Darius, Stadler Michael B., Hadziselimovic Faruk (2017). DMRTC2, PAX7, BRACHYURY/T and TERT Are Implicated in Male Germ Cell Development Following Curative Hormone Treatment for Cryptorchidism-Induced Infertility. Genes.

[47] Hadziselimovic Faruk, Cathomas Gieri, Verkauskas Gilvydas, Dasevicius Darius, Stadler Michael (2018). PRDM Histone Methyltransferase mRNA Levels Increase in Response to Curative Hormone Treatment for Cryptorchidism-Dependent Male Infertility. Genes.

[48] Gao N, Zhang J, Rao MA, Case TC, Mirosevich J, Wang Y (2003). The role of hepatocyte nuclear factor-3 alpha (Forkhead Box A1) and androgen receptor in transcriptional regulation of prostatic genes. Mol Endocrinol.

[49] Huang KB, Zhang SP, Zhu YJ, Guo CH, Yang M, Liu J (2019). Hotair mediates tumorigenesis through recruiting EZH2 in colorectal cancer. J Cell Biochem.

[50] Aiello A, Bacci L, Re A, Ripoli C, Pierconti F, Pinto F (2016). MALAT1 and HOTAIR long non-coding RNAs play opposite role in estrogen-mediated transcriptional regulation in prostate cancer cells. Sci Rep.

[51] Cheng Y, Jutooru I, Chadalapaka G, Corton JC, Safe S (2015). The long non-coding RNA HOTTIP enhances pancreatic cancer cell proliferation, survival and migration. Oncotarget.

[52] Watanabe M, Fukuda A, Nabekura J (2014). The role of GABA in the regulation of GnRH neurons. Front Neurosci.

[53] Berghoff EG, Clark MF, Chen S, Cajigas I, Leib DE, Kohtz JD (2013). Evf2 (Dlx6as) lncRNA regulates ultraconserved enhancer methylation and the differential transcriptional control of adjacent genes. Development..

[54] Kaewman P, Nudmamud-Thanoi S, Thanoi S (2018). GABAergic alterations in the rat testis after methamphetamine exposure. Int J Med Sci.

[55] Lo Iacono N, Mantero S, Chiarelli A, Garcia E, Mills AA, Morasso MI (2008). Regulation of Dlx5 and Dlx6 gene expression by p63 is involved in EEC and SHFM congenital limb defects. Development..

[56] Bhattacharyya S, Bronner-Fraser M (2008). Competence, specification and commitment to an olfactory placode fate. Development..

[57] Ji Qing, Liu Xuan, Fu Xiaoling, Zhang Long, Sui Hua, Zhou Lihong, Sun Jian, Cai Jianfeng, Qin Jianmin, Ren Jianlin, Li Qi (2013). Resveratrol Inhibits Invasion and Metastasis of Colorectal Cancer Cells via MALAT1 Mediated Wnt/β-Catenin Signal Pathway. PLoS ONE.

[58] Wang Jun, Zhang Xuan, Chen Wen, Hu Xiang, Li Jing, Liu Changning (2019). Regulatory roles of long noncoding RNAs implicated in cancer hallmarks. International Journal of Cancer.

[59] Pu H, Zheng Q, Li H, Wu M, An J, Gui X (2015). CUDR promotes liver cancer stem cell growth through upregulating TERT and C- Myc. Oncotarget..

[60] Ye Y, Li L, Zheng Z, Chen S, Chen E, Hu Y (2017). Long non-coding RNA linc00261 suppress gastric cancer progression via promoting slug degradation. J Cell Mol Med.

[61] Ko S, Ahn J, Song CS, Kim S, Knapczyk-Stwora K, Chatterjee B (2011). Lysine methylation and functional modulation of androgen receptor by Set9 methyltransferase. Mol Endocrinol.

